# Research Progress of NMR in Natural Product Quantification

**DOI:** 10.3390/molecules26206308

**Published:** 2021-10-19

**Authors:** Zhi-Fan Wang, Yu-Lin You, Fei-Fei Li, Wen-Ru Kong, Shu-Qi Wang

**Affiliations:** School of Pharmacy Sciences, Shandong University, 44# West Wenhua Road, Jinan 250012, China; wangzhf_sunset@163.com (Z.-F.W.); youyulin@mail.sdu.edu.cn (Y.-L.Y.); lifeifei2013@126.com (F.-F.L.); qfkongwenru@163.com (W.-R.K.)

**Keywords:** quantitative nuclear magnetic resonance (qNMR), natural products, quantitative analysis

## Abstract

In the fields of medicine and health, traditional high-performance liquid chromatography or UV-visible spectrophotometry is generally used for substance quantification. However, over time, nuclear magnetic resonance spectroscopy (NMR) has gradually become more mature. Nuclear magnetic resonance spectroscopy has certain advantages in the quantitative analysis of substances, such as being nondestructive, having a high flux and short analysis time. Nuclear magnetic resonance spectroscopy has been included in the pharmacopoeiae of various countries. In this paper, the principle of nuclear magnetic resonance spectroscopy and the recent progress in the quantitative study of natural products by NMR are reviewed, and its application in the quantitative study of natural products is proposed. At the same time, the problems of using NMR alone to quantify natural products are summarized and corresponding suggestions are put forward.

## 1. Introduction

Nuclear magnetic resonance (NMR) is a commonly used analytical technique. In 1945, the two groups led by Bloch and Purcell discovered the NMR phenomenon at almost the same time. Bloch [1] and Purcell [2] simultaneously demonstrated NMR in condensed matter (water and paraffin, respectively). Their work has enabled NMR to be used not only in molecular beams but also in liquids and solids, and the application of NMR had begun. NMR refers to the phenomenon where the energy level of nuclei with spin split under the action of an external magnetic field, and energy level transitions can occur with a specific radio frequency radiation. In the field of chemistry, NMR is used in structural analysis and quantitative analysis. However, because the early technical conditions were not sufficiently advanced and since the degree test method verification was poor, its application was limited to structural determination. Since the 1970s, the commercialization of pulsed Fourier transform NMR spectroscopy, advances in high-field magnets, pulse trains, and cryo probe techniques have led to significant improvements in the sensitivity and resolution of NMR quantitative analysis methods [3]. At present, NMR technology is increasingly widely used in the fields of food science [4,5,6], medicine and health [7,8,9,10,11], organic chemistry [12], metabolomics [13,14,15], animal and plant microbes [16,17], and natural products [18].

In the past, we have summarized relevant reviews, including those discussing the content determination of complex mixtures [19], biological metabolites [20], natural samples [21,22,23,24], drugs [7,25,26,27], and traditional Chinese medicine samples [28]. Among them, the review “Quantitative H-1 NMR: Development and potential of a method for natural products analysis” is relatively comprehensive and highly cited in this field, which provides many valuable ideas for our writing.

This review will focus on the quantification of natural products by NMR. Natural products have complex and diverse ingredients, which contain many active compounds, and are valuable resources of drug candidates and drug scaffolds. Since the composition of natural products is more complex than that of synthetic chemical products, it is necessary to use a large number of reference substances when using traditional chromatographic and spectral methods for their qualitative and quantitative analysis. However, due to the particular composition of natural products, few corresponding reference substances are available, which increases the cost of quantitative tests for many natural products [22]. Compared to other quantitative analysis methods, NMR generally uses liquid samples. When the impurity peaks do not interfere with the sample peak or when the impurities do not have peaks, quantitative NMR is not concerned with sample impurities, does not require standard reference materials, and the entire process will not cause damage to the sample, which makes it extremely applicable for the quantitative testing of natural products.

Quantitative NMR technology covers a variety of nuclei, such as ^1^H, ^13^C, ^19^F, ^17^O, ^31^P, etc. However, to date, ^1^H NMR is the most widely used because of its higher sensitivity and satisfactory quantitative relationship. At present, the minimum error range can be controlled within 2% [22]. For the sake of convenience and through literature review, “qNMR” is used as a general abbreviation of quantitative NMR in the following sections.

**Figure 1:**
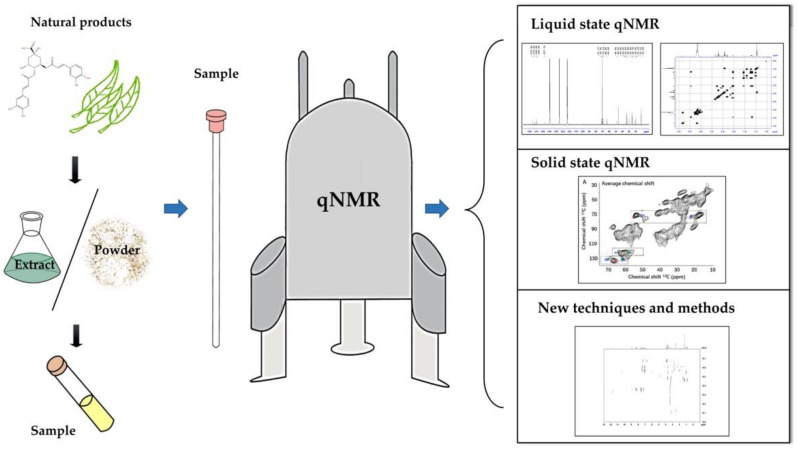
The graphical summary.

Below is a graphic summary of the structure of the review (Figure 1).

## 2. Overview of qNMR

### 2.1. Basic Principles

There are four main parameters in NMR, namely chemical shifts, peak areas, coupling constants, and relaxation times. Chemical shifts and coupling constants are important parameters for material structure elucidation, while peak areas and peak heights are the main basis of material quantification. Since the relaxation time of the hydrogen spectrum are relatively short, it generally does not affect the resolution of the peak areas of the spectrum.

**Figure 2:**
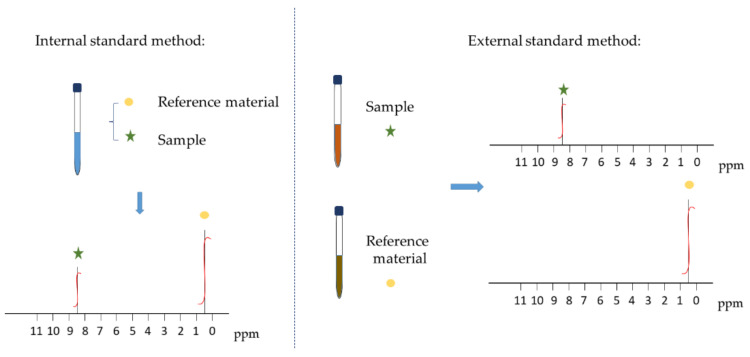
Internal standard and external standard method.

The basic principle of qNMR is to determine the number of protons at each position in the molecule from the integral area and to quantify it according to the amount of substance in the analyte that is proportional to the integral area of the corresponding resonance peak. There are two basic modes: absolute quantification and relative quantification [20], and the absolute quantitative method is often used in practice. Absolute quantification includes the internal standard method, external standard method, and standard substance addition method, among which the internal standard method is commonly used (Figure 2). One advantage of the internal standard method is that a standard curve is not necessary.

Fernandez-Pastor et al. [29] used the internal standard method to quantify L-Dopa in *M. Pruriens* seeds, and an NMR calibration curve was obtained to evaluate the accuracy and reproducibility of the method. The results confirm that the internal standard method has reliable accuracy and reproducibility and that a standard curve is not needed for the quantification. Of course, natural products that have corresponding reference materials can also be directly quantified by the external standard method. Choudhary et al. [30] used the external standard method to quantify the major sesquiterpene lactones in the essential oil of *Inula Racemosa* and *Saussurea Iappa*. In the internal standard method, compounds with a known structure are selected as internal standard substances. The sample to be tested needs to be precisely weighed, as does the the internal standard substance of known purity, and then the solution is prepared for testing using NMR analysis. The content of the sample to be measured can be calculated by the peak area ratio. The calculation formula [28] is as follows:

$$\mathrm{Px}=\frac{Ix}{Istd}\frac{Nstd}{Nx}\frac{Mx}{Mstd}\frac{mstd}{mx}Pstd$$

In the formula, Px and *Pstd* represent the purity of the object to be measured and the internal standard, *Ix* and *Istd* represent the peak area of the object to be measured and the internal standard, *Nx* and *Nstd* represent the number of hydrogen atoms corresponding to the quantitative peak in the object and the internal standard, *mx* and *mstd* represent the molar mass of the object to be measured and the internal standard, and *Mx* and *Mstd* represent the mass of the object to be measured and the internal standard.

In addition to the quantitative methods introduced above, another quantitative method is becoming more and more popular: quantification with electronic calibration. In 1997, Barantin and Akoka [31,32] introduced an absolute quantitation method for drug concentrations—electronic reference to access in vivo concentrations (ERETIC). In this method, a free coil is arranged in the carbon head, which can generate a reference signal during sampling. The accurate chemical displacement and integral value can be obtained by controlling the amplitudes, phases, and frequencies of the signal, and then the content of the compounds can be measured. The ERETIC method can be used with all nuclei and all spectrometers because all of the parameters of the reference signal are easily adjustable over a wide range. There is a large amount of literature [33,34,35,36] that shows that this method has more advantages than traditional internal and external standard methods, especially for the quantification of metabolites in vivo. At present, this method has been applied in ^1^H [37], ^13^C, and 2D NMR [38]. Quite a few studies in the literature show that this method has more advantages than traditional internal and external standard methods, especially for the quantification of metabolites in vivo.

### 2.2. The Choice of Internal Standards

When the internal standard method is used for quantitative NMR, an appropriate internal standard is needed. As an ideal internal standard, it should meet the requirements of having stable physical and chemical properties, good solubility in deuterated reagents, high purity, few signal peaks, and no overlap with the quantitative peaks in the analyte. Commonly used internal standard substances are aromatic rings, alkanes, silanes, and other compounds. Torgny et al. conducted tests on 25 candidate internal standards and finally selected eight suitable internal standards. They are 2,4,6-triiodophenol, 1,3,5-trichloro-2-nitrobenzene, 3,4,5-trichloropyridine, dimethyl terephthalate, 1,4-dinitrobenzene, 2,3,5-triiodobenzoic acid, maleic acid, and fumaric acid [39]. In addition, studies have shown that it is also a good choice to directly use the residual solvent signal as an internal standard. Gregory K et al. [40] used the residual solvent dimethyl sulfoxide (DMSO-d_6_) as an internal standard for quantitative analysis, and Ruslan et al. [41] showed that the residual solvent deuterated chloroform (CDCl_3_) could also be used as an internal standard for direct quantitative analysis. The method of using residual solvent signal as an internal standard significantly simplifies sample preparation.

### 2.3. Main Types

According to the physical state of the sample, qNMR techniques can be divided into liquid qNMR and solid qNMR, of which the most commonly used is liquid NMR. However, some samples cannot be dissolved in any solvent, or dissolution in the solvent will cause changes in their crystal type, such as some polymers, natural samples such as plants [42,43] and soil [44]; biological structure samples [45,46]; drugs with a certain crystal type [47,48,49]; and additives in solid food [50], etc. Solid-state nuclear magnetic resonance techniques are the preferred method for the qualitative and quantitative analysis of such samples. The following sections will use “ssNMR” as an abbreviation for solid-state nuclear magnetic resonance technology.

NMR can be divided into one-dimensional, 2D, 3D, and 4D NMR. Commonly used one-dimensional methods include ^1^H, ^13^C, DEPT, and NOE-diff spectrum. Two-dimensional spectra include COSY, HMQC/HSQC, TOCSY, and NOSEY/ROESY. In recent years, three-dimensional and four-dimensional NMR techniques have been used to confirm the structure of matter. Since this paper is mainly concerned with the quantification of natural products, they will not be discussed below.

## 3. Advantages of qNMR

The composition of most natural products is complex, and traditional quantitative methods are no longer advantageous for their quantitative determination. For example, the nine salvianolic acids in the salvianolate lyophilized injection (SLI) have such highly similar structures, amd traditional HPLC struggles to analyze its contents. Chen et al. [51] applied qNMR to 10 batches of SLI, and the results were very impressive. Liu et al. [52] quantified the active components in *Artemisia annua* by qNMR using maleic acid as the internal standard and compared the qNMR test results with conventional quantitative techniques. The test results were basically consistent with those determined by HPLC-ELSD and LC-MS. Chauthe et al. [53] applied NMR to the medicinal plants *Eugenia Jambolana*, *Withania Somnifera,* and *Aegle Marmelos* and their herbal products. The samples were dissolved in deuterated solvents, and 1,3,5-trimethoxybenzene (TMB) was used as an internal reference standard. The qNMR results were in accordance with the HPLC results, suggesting that qNMR is as reliable as HPLC for quantification analysis. Freitas et al. [54] applied GC-MS, GC-FID, and ^1^H NMR to determine the essential oil content in *O. basilicum* L. and *O. gratissirnum*. Despite the corroborating results, the chemometric analysis revealed the higher robustness (better adjustment) of the ^1^H NMR model compared to the GC-MS method in terms of certain statistical parameters. All of the above studies demonstrate the advantages of qNMR for the quality control of complicated natural products: simple sample preparation and high efficiency and reliability.

At the same time, more studies have shown that qNMR has obvious advantages in analyzing Chinese herbal medicine. Fan et al. [55] used qNMR to determine the content of six alkaloids (berberine, jatrorrhizine, epiberberine, coptisine, palmatine, and columbamine) in *Rhizoma coptidis* from three different habitats. The recoveries ranged from 96.6% to 102.4%. The results showed that the qNMR method was a reliable method to evaluate the quality of Chinese herbal medicine containing berberine. Li et al. [56] and Huang et al. [57] used qNMR to determine the content of ginsenoside, proving that qNMR is a valuable and reliable method of the qualification assessment of the composition at low purities, particularly for the products derived from plants or herbal medicines. Furthermore, Yu et al. [58] applied qNMR to the quantification of resveratrol and polydatin in *Polygonum Cuspidatum* traditional Chinese herb cuts and granules. Bertelli et al. [59] applied NMR to the quantification of both prenylflavonoids and bitter acids in eight hop cultivars, which is more effective than traditional HPLC.

Up until now, much of the literature has pointed out the high accuracy and reproducibility of qNMR, and some of the results are comparable to classical HPLC-UV, GC-MS, LC-MS, and other techniques.

## 4. Research Progress of qNMR Applied to Natural Products

After many years of development, qNMR has been widely used in the examination of impurities and for the content determination of Chinese herbal medicine and natural products [60]. Kuchta et al. [61,62] studied stachydrine using ^1^H qNMR for two consecutive years and compared the results with those obtained by traditional methods. The results show that qNMR is reliable in material quantification. The application of quantitative nuclear magnetic technology in natural products will be summarized below.

### 4.1. Liquid State qNMR

#### 4.1.1. ^1^H qNMR

NMR was initially widely used for the qualitative analysis of organic compounds, mainly to elucidate the structure of unknown compounds. Later, it was gradually applied to the quantification of matter. For a mixture system, such as natural substrates, if each component can find a hydrogen peak group that does not overlap with the other components and fully consider the solvent or macromolecular component signal suppression and spiking [63], we can use the hydrogen spectrum for quantitative work. Thus, when using ^1^H qNMR, attention should be paid to the adjustment of relevant experimental parameters during quantitative experiments and the corresponding complex technology should be used to suppress this kind of signal. Unless there is only one NMR peak, it is usually not accurate to quantify the substance only by the peak signal strength. Xiao et al. [64] divided the methods of solvent suppression in liquid NMR experiments into six categories, among them, CPMG (Carr–Purcell–Meiboom–Gill) uses the characteristic that the transverse relaxation time T2 of the solvent signal is shorter than that of the solute to suppress the solvent peak. By selecting the appropriate echo parameters, macromolecules with short T2, which have broad signals, can be attenuated or even eliminated from the spectrum [65]. According to Aguilar et al., pre-saturation and the Carr–Purcell–Meiboom–Gill (Presat–CPMG) pulse sequence is the most commonly used solvent suppression method, but it has some defects, such as imperfect suppression of the water signal, large radio-frequency power demands, and decreasing performance with increasing magnetic field [66]. Therefore, Aguilar et al. proposed new pulse sequences and pointed out that CPMG pulse sequences can be replaced with WASTED pulse sequences. In addition, Pauli et al. [22] put forward two views on this issue: one is quantitative experimental conditions, including appropriate parameter selection such as relaxation delay, digitization, and pulse sequence design, and another is the selection of appropriate post acquisition processing parameters for optimized spectral integration.

^1^H NMR, as the most commonly used NMR analysis method, has been widely used for the quantitative determination of a specific natural product in a variety of complex mixtures. We have searched the relevant literature from the past 10 years and have listed the application examples of ^1^H NMR in the form of tables for reader reference. Table 1 summarizes literature on the application of ^1^H qNMR to the content determination of natural products. In the literature, the accuracy of ^1^H qNMR has been fully verified.

#### 4.1.2. ^13^C qNMR

In general, ^13^C NMR is used for structural confirmation and less for quantification due to the total hydrogen decoupling of carbon. Different types of carbon signals are correspondingly different. Therefore, the use of ^13^C in the quantification of substances is limited. However, the quantitative work of ^13^C can also be achieved by using standard curve and other methods. The respective ^13^C NMR spectra obtained under proton decoupling are spread over a larger chemical shift range relative to the ^1^H NMR spectrum, and the resolution of the spectra is generally higher. Quantitative ^13^C NMR spectroscopy requires a longer relaxation delay, which may result in a longer acquisition time needed to achieve a sufficient signal-to-noise ratio. In addition, ^13^C has low sensitivity due to the low isotopic abundance in comparison to ^1^H and ^13^C signal intensity, which is affected by the nuclear overhauser Enhancement (NOE) effect. Based on this, Marchetti et al. [103] put forward corresponding solutions for reader reference, such as concentrating the samples, measuring the T_1_ for each carbon of standard compounds tentatively, and lowering the proton decoupling power to zero. With the continuous improvement of methods, ^13^C NMR has been better used for quantitative work on substances.

Marchetti et al. [103] developed a reliable ^13^C qNMR method for the multi-component analysis and determination of the main non-psychoactive cannabinoids (cannabidiol, cannabidiolic acid, cannabigerol, and cannabigerolic acid) in female inflorescences of different *hemp* varieties. In comparison with the most commonly HPLC technique, this qNMR approach was precise and sufficiently sensitive. Colombo et al. [104] used ^13^C qNMR to quantitatively determine the relative fructose isomer composition and total fructose content in wine. It was found that this method is also suitable for the detection and quantification of substances that have at least one ^13^C signal and that do not overlap with the resonance of other compounds. Duquesnoy et al. [105] developed a procedure using ^13^C qNMR spectroscopy, which allowed for the direct identification and quantification of the carbohydrates in the ethanol extracts of *Pinus nigra Arnold* ssp. *Laricio Poiret* and *Abies alba Miller*, which avoids multi-step sample preparation before analysis. Kazalaki et al. [106] applied a methodology based on ^13^C NMR spectroscopy to detect and quantify fourteen mono-, di-, and trisaccharide molecules in authentic Greek honey samples and strictly verified this analytical method (accuracy, linearity, range, limit of detection, etc.).

#### 4.1.3. 2D qNMR

One-dimensional nuclear magnetic resonance techniques are simple to operate and have the same sensitivity and accuracy as traditional chromatography and take less time. However, when the sample is a complex mixture, the spectral peak of one-dimensional NMR has a high overlap, and the spectral resolution decreases accordingly. The spectral resolution is inefficient or even impossible to complete. Two-dimensional NMR usually appears as a powerful analytical means of identifying unknown compounds. Two-dimensional NMR solves the overlap problem, but at the cost of biasing peak intensities [107]. This is partly due to the uneven coherence transfer between excited/detected ^1^H nuclei and the heteronuclei coupled to them (typically ^13^C); at the same time, 2D NMR has generally been limited to qualitative measurements due to its high sensitivity to pulse imperfections and instrumental requirements [108].

Although COSY is the most commonly used 2D NMR, technique HMQC and HSQC have more advantages in the analysis of complex compounds in the absence of complete data. HMQC associates the carbon spectrum and hydrogen spectrum of a compound, revealing the carbon connection relationship by combining the COSY and HMQC or HSQC spectra [109]. Two-dimensional NMR can be used in combination with traditional analytical chemical methods to deduce the structure of unknown compounds and is the cornerstone of modern structure elucidation methods [110]. Many researchers have thus discovered a variety of new substances [111,112,113,114,115,116,117,118] that can be used to develop new drugs or in biological research.

In contrast to one-dimensional qNMR, two-dimensional qNMR is an indirect quantification method and needs suitable standard compounds for calibration. Serhat et al. [119] chose a suitable substance as the surrogate standard to quantify the anthraquinones in the bark of *alder buckthorn (Frangula alnus),* and 2D NMR was validated for its accuracy, precision, specificity, linearity, and limit of quantitation. Hu et al. [120] illustrated the application of the HSQC approach in the selective quantification of thiocoraline. Calibration curves of eight different NMR cross-peaks were established using dimethyl terephthalate as an internal standard. Serhat et al. [121] described the quantitation of eight diterpene acids in the oleoresin of *Copaifera reticulata Ducke*, and the results showed that the qNMR method had better precision in the quantification of the three most abundant compounds. Additionally, Serhat et al. [122] used band-selective quantitative HSQC and quantitative H, H COSY for the selective quantification of sweet-tasting 11-α-hydroxy-mogrosides in *Siraitia grosvenorii* fruits. The results were satisfactory. In addition, the COSY experiment has advantages in terms of its accuracy, precision, and quantitation limit.

Meanwhile, 2D NMR alone may be limited, and it has two significant drawbacks [123]. First, the 2D NMR signal is strongly molecule-dependent and site-dependent; second, the duration of 2D NMR is so long that prevents its general use for high-throughput quantitative applications and affect the quantitative performance. Moreover, 2D spectra are usually sensitive to spectrometer instabilities, and 2D NMR is sometimes not suitable for unstable samples [124]. In 1D qNMR, the peak area is directly proportional to analyte concentration, allowing for accurate and precise quantitation, but the intensities of cross peaks in standard 2D NMR experiments are not directly proportional to analyte concentration due to resonance specific signal attenuation during the pulse sequence, so Le et al. [125] highlighted the advantages of a combination of 1D and 2D quantitative NMR to accurately assess the amounts of alkaloids with different ranges of concentrations and stability within extracts.

### 4.2. Solid State qNMR

Although ssNMR is produced at almost the same amount of time as the traditional NMR technology, the wide spectral lines generated by ssNMR, if no corresponding measures are taken, are not beneficial to the qualitative or quantitative analysis of substances. Besides this, ssNMR is expensive and has long analysis times as well as low throughput [126], so, for a long time, ssNMR developed slowly. Until the 1980s, due to the development of the NMR spectrometer and related technologies, ssNMR further evolved [127]. In the early 21st century, ssNMR gradually matured, and an increasing number of researchers began to regard ssNMR as an effective means of quantifying solid substances [128]. Li et al. [129] took drug substances, excipients, and solid dosages as an example and summarized the application of ssNMR in drug production.

Most ssNMR experiments are used in the quantitative determination of biological macromolecules, such as proteins, and biological metabolites, etc. [46]. Narasimhan et al. [130] studied the changes in the target proteins in their native setting using ssNMR. In recent years, amyloid research has promoted the development of some novel drugs. The application of ssNMR provides new impulses in the understanding of the role of the amyloid fold in native biological functions [131,132,133].

Lignin is a kind of complex organic polymer, most of which exists in the cell wall of plants. As a natural product, lignin is widely used in industry. Due to its extremely complex composition, the extraction and separation process of lignin is often complicated. Gao et al. [134] used ^13^C CP/MAS NMR to directly determine lignin structures without destroying its own structure. In addition to lignin, the cell walls in plants and microbes are rich in certain carbohydrate materials. Bernardinelli et al. [135] used quantitative ^13^C MultiCP ssNMR to study the effects of consecutive acid/alkaline pretreatments and enzymatic hydrolysis on sugarcane bagasse; the results revealed that this method can be used to efficiently estimate the crystallinity index (CI) of the cellulose inside the intact biomass. Fernando et al. [136] summarized the recent ssNMR studies that have elucidated the polymorphic structure and heterogeneous dynamics of polysaccharides and other biomolecules, such as proteins, lignin, and pigment, in the intact cell walls or biofilms of 11 species across plants, fungi, bacteria, and algae.

Zhu et al. [137] presented a new method utilizing ^13^C CP/MAS NMR spectra for the simultaneous quantitative determination and structure analysis of pectin in tobacco; the results were in good agreement with the conventional chromatographic method.

### 4.3. New Techniques and Methods

As a general detection technology, NMR has significant advantages in many aspects, from liquid NMR to solid NMR, from one-dimensional to two-dimensional, from universal hydrogen core, and from carbon core to heteroatoms applications.

The first advancement is the application of heteroatom NMR such as ^19^F, ^17^O, ^31^P. Some natural products and proteins contain phosphorus, the spectra of which are relatively simple. Bhinderwala et al. [138] incorporate ^31^P NMR into a metabolomics investigation. However, the lines of ^31^P NMR spectra tend to be wide, and ^31^P NMR has not been widely used. Wang et al. [139] developed a novel and sensitive dispersive liquid–liquid micro-extraction combined with ^19^F qNMR methods for the determination of fipronil and its metabolites in chicken eggs, which has been proven to have high operability and accuracy.

The second progression is the application of multidimensional NMR. Multidimensional NMR is an effective solution to the problem of quantitative peak overlap in NMR. In recent years, the application of fast 2D NMR technology is also common. Due to the time-consuming property of 2D NMR technology, it is difficult to use for high-throughput analysis. An increasing number of researchers have begun to pay attention to the development of fast NMR technology. Sette et al. [140] adopted rapid quantitative HSQC (QQ-HSQC) using guaiacyl C2 and syringyl C2–C6 signals as internal standards and quantitatively evaluated a series of different inter-units of lignin. In 2013, Martineau et al. [141] proposed three strategies, including spectral aliasing, linear prediction, and nonuniform sampling, in order to shorten the duration of heteronuclear 2D NMR. However, the last strategy was proven to lead to precision losses. Girreser et al. [142] applied band-selective quantitative heteronuclear single-quantum correlation spectroscopy (bs-qHSQC) for the quantification of aloin and total anthranoids in *Aloe* vera and *Aloe* ferox, which is comparable to standard methods in terms of precision and accuracy. In addition, a method was established and was used to determine the ratio of the two diastereomers of aloin A and B. Le Guennec et al. [143] summarized the advantages and disadvantages of multi-dimensional NMR and proposed that one of the main research challenges is to reduce the time of multi-dimensional NMR experiments. Rapid 2D technology has the potential to obtain spectra in shorter acquisition times in the quantitative analysis of complex mixtures. Giraudeau detailed a decade-long effort to achieve high accuracy and precision in two-dimensional NMR quantitative measurements, with the introduction of the Ultrafast COSY and zTOCSY in 2011 [144]. Gouilleux et al. [145] highlights the numerous developments that had occurred until 2017, and the ultrafast (UF)NMR can be used as a highly versatile quantitative NMR method.

In their review, Huang et al. [146] classified the advanced qNMR approaches into three categories: a combination of qNMR and chromatography, advanced spectroscopic techniques for qNMR, and chromatography with qNMR-assisted SI-traceable calibration. These new, advanced methods could minimize the effects of impurities and improve the applicability of previous methods. Coulibaly et al. [147] developed a qNMR method for real-time quantification, providing convenience for the real-time monitoring of contents. Golowicz et al. [107] presented a novel approach named swept coherence transfer (SCoT), which is based on the idea of time-resolved NUS acquisition in HSQC experiments; the coherence transfer delay is co-incremented with non-uniformly sampled evolution time. They performed the measurements on a mixture of 17α-ethynlestradiol and m-anisaldehyde; it was found that SCoT can solve the main problem, limiting the use of 2D NMR to quantitative chemical analysis, and can greatly reduce the disturbances of peak intensities and phases, contrary to conventional experiments. The speed advantage is more obvious when used in multidimensional experiments.

Due to the highly similar structure of the isomeric components, an ineluctable spectral feature of isomeric mixtures is the heavy overlap of signals in 1D and 2D NMR spectra. Yang et al. [148] proposed a new 1D selective NMR experiment, chemical shift selective filter (CSSF) -TOCSY-INEPT, which allows the extraction of discrete isomers of the ^13^C NMR subspectra in complex mixtures without physical separation.

In practice, the simple use of NMR encounters a variety of problems, such as low selectivity, impurity interference bias, etc. Therefore, upgrades and improvements based on the original technology have become a hot topic of current discussion. Only the continuous innovation of technology and methods can better demonstrate the advantages of NMR.

## 5. Latest Development of qNMR

In the 21st century, both liquid and solid NMR technology are developing rapidly and are favored by an increasing number of scholars. Middleton [149] considered the ssNMR technique a promising counterpart to the conventional experimental techniques of X-ray crystallography and solution-state NMR to provide the structural features of drug targets that can guide medicinal chemistry towards drug candidates. With the development of 3D and 4D technology, NMR technology provides a higher level of convenience and the possibility of molecular biology research [150].

### 5.1. Shortcomings and Improvements

Although NMR has many advantages when used for material quantification, one of its disadvantages is low sensitivity. In the past two decades, in order to overcome this low sensitivity, Bruker introduced Cryo and Micro diameter probe-head technology and developed new hardware and software [151], making NMR data-processing easier and faster. For complex samples with a low concentration, there is often uncertainty in quantification, so the samples need to be treated accordingly [78,152]. Liu et al. [153] used solid phase extraction (SPE) to extract the epigoitrin in Radix Isatidis decoction pieces and increased the detection sensitivity. Similarly, Liu et al. [154] used the SPE method to extract icariin in Ruzengning capsules prior to quantitative analysis by NMR.

Moreover, one drawback of ssNMR quantification is the high demand for samples; however, many natural products are difficult to obtain in quantities greater than a few milligrams [49]. Fan et al. [155] provide a solution to the problem of sample requirement by referring to some methods of sample preparation in liquid NMR (protein isotope labeling to produce inexpensive samples).

### 5.2. Combination with Other Quantitative Techniques

The combination of NMR and traditional analytical methods is also an important direction for future research. The concentration of qNMR tests has a certain threshold value, so its combination with more sensitive analytical techniques is generally selected (e.g., HPLC, MS). The combination of multiple analytical methods is more suitable for metabolomics research [14,24]. Watson et al. [156] used both NMR and mass spectrometry as effective tools to determine the degree of acetylation of chitin in Penicillium Chrysogenum. Lu et al. [157] combined NMR with biological methods to evaluate the quality of Chinese medicinal materials. Masumoto et al. [158] combined ^1^H qNMR and an HPLC/photodiode array (PDA) detector (or an HPLC/variable-wavelength detector (VWD)) to calculate the exact relative molar sensitivity (RMS) and employe single-reference DFS with RMS to determine the content of perillaldehyde. Finally, with the development of instruments, the upgrading of data-processing systems and the improvement of experimental methods, the application of qNMR will enter a new stage.

## 6. Conclusions

Compared to traditional chemical analysis methods, as an advanced chemical analysis method, qNMR has several advantages, which make it widely used in various fields; it is especially suitable for the determination of complex natural products in Chinese herbal medicine. Its principle is simple, and the method is easy to operate. In addition, no damage to the samples occurs during the analysis of the samples. Finally, its reproducibility and accuracy are comparable to the traditional chemical analysis methods. Based on the mature development of traditional liquid NMR technology, the application of solid NMR technology is also common. The development of solid NMR technology provides a new idea for the analysis of solid samples, and the processing process of samples has been further simplified. Meanwhile, NMR also has shortcomings, such as its low sensitivity, inability to quantitatively analyze trace substances, and long analytical spectrum. However, we believe that NMR will gradually become a mainstream quantitative analysis method with its continuous improvement or in combination with the traditional analysis methods in series.

## Figures and Tables

**Table 1:** Natural products determined by nuclear magnetic quantitative technique.

Object of Study	Quantified Component	Internal Standard	Deuterated Solvent	Reference
*Artemisia annua*	artemisinin	maleid acid	Methanol-*d_4_* (CD_3_OD)	[52]
*Black cohosh (Actaea racemosa)* rhizomes	cycloartanoids	1,2,4,5-tetrachloro-3-nitrobenzene	DMSO-*d_6_*, CD_3_OD	[67]
*Ginkgo biloba* L.	terpene trilactone components	HMDSO	CD_3_OD, benzene-*d_6_*	[68]
*I. aromatica and I. henryi.*	actinodaphnine	1,4-dinitrobenzene	DMSO-*d_6_*	[69]
three species of *Rhodiola*	the metabolites of rhodiola	3-(trimethylsilyl) propionic-2, 2, 3, 3-*d_4_* (TMSP)	CD_3_OD, deuterium oxide (D_2_O), CDCl_3_, DMSO-*d_6_*	[70]
*Angelicae Pubescentis Radix*	osthol, isoimperatorin, and columbianadin	pyrazine	DMSO-*d_6_*	[71]
*Danshen*	22 metabolites	tetramethyl silane (TSP-*d_4_)*, TMSP	D_2_O, CD_3_OD	[72]
*Salvia mltiorrhiza Bunge*	Tanshinone I, tanshinone IIA, dihydrotanshinone, and cryptotanshinone	3,4,5-trichloropyridine	CDCl_3_	[73]
*Paederia foetida*	scopoletin	TSP-*d_4_*	CD_3_OD, CDCl_3_, DMSO-*d_6_*, TSP-*d_4_*	[74]
*Angelica a dahurica*	imperatorin, byakangelicin, and oxypeucedanin	hydroquinone	DMSO-*d_6_*	[75]
*Angelicae sinesis*	ferulic acid, coniferyl ferulate, and ligustilide	pyrazine	DMSO-*d_6_*	[76]
Shuanghuanglian capsule	chlorogenic acid	1,4-phthalaldehyde	DMSO-*d_6_*	[77]
*Angelicae Pubescentis Radix*	osthole, columbianadin, and isoimperatorin	pyrazine	DMSO-*d_6_*	[78]
*Myrcia multiflora (Lam.) DC*.	6 extract phenolic compounds	TMSP	CD_3_OD	[11]
*Ligusticum chuanxiong Hort*	Z-ligustilide and senkyunolide A	1,4-dinitrobenzol	CDCl_3_	[79]
*Moutan* *cortex, Hachimijiogan, Keishibukuryogan*	Paeonol	Hexamethyldisilane (HMD)	CD_3_OD	[80]
Anxiolytic fraction of *Juncus effusus* L.	phenanthrenes	terephthalic acid	DMSO-*d_6_*	[81]
*Fraxinus angustifolia, Fraxinus ornus*	acteoside	4,4-dimethyl-4-silapentane-1-sulfonic acid (DSS)	CD_3_OD	[82]
*Rhizoma Coptidis*	berberine, coptisine, jatrorrhizine, palmatine, and epiberberine	Electronic reference	CD_3_OD	[37]
breviscapine preparations	scutellarin	TSP-*d_4_*	DMSO-*d_6_*	[83]
*Hibiscus sabdariffa*	metabolites in different hibiscus teas	TSP-*d_4_*	CD_3_OD	[84]
*Lycium*	betaine	maleic acid, succinic acid	D_2_O	[85]
essential oils of *Eremanthus erythropappus*	α-bisabolol	Octamethylcyclotetrasiloxane (OMCTS)	CDCl_3_	[86]
oils of *eucalyptus, pink pepper and turpentine*	α-pinene	OMCTS	CDCl_3_	[87]
*Capparis spinosa* L. root	stachydrine	maleic acid	D_2_O	[88]
*Xinjiang licorice*	licochalcone A, licochalcone B, glabrone, echinatin	1,4-bis(trimethylsilyl)benzene-d4 (1,4-BTMSB-d4)	Acetone-*d6*	[89]
wheat malt beer	6-methoxy-2-(3*H*)-benzoxazolone	syringaldehyde	CDCl_3_	[90]
*Entandrophragma angolense*	23- *O*-deethylanderolide S.	residual solvent	CDCl_3_	[91]
*Hypochaeris radicata*	4-(3,4-dihydroxybenzyl)-2-(3,4-dihydroxyphenyl) tetrahydrofuran-3-carboxy-*O*-β-D-glucopyranoside, hypochoeroside C, *hypochoeroside D*, 5-*O*-caffeoylshikimic acid	Dimethyl terephthalate	DMSO-*d_6_*	[92]
*Actaea racemosa, A. podocarpa, A. cordifolia*	cycloartane triterpenes	residual solvent signal (DMSO-*d_5_*)	DMSO-*d_6_*	[93]
*Coffea canephora var. robusta beans*	16-*O*-methylcafestol and kahweol	*N*, *N*-dimethylformamide (DMF)	D_2_O	[94]
*Camellia sinensis* (L.) *Kuntze*	green tea catechins	TMS	DMSO-*d_6_*	[95]
*Trifolium pratense* L.	isoflavones	3,5-Dinitrobenzoic acid internal calibrant	DMSO-*d_6_*	[96]
*Crataegus monogyna, C. laevigata, C. douglasii, C. okanaganensis*	naringenin, hyperoside, rutin, vitexin-2″-*O*-rhamnoside	DSS	CD3OD	[97]
*Turmeric* oleoresin	curcuminoids	TSP-*d_4_*	DMSO-*d_6_*, Acetone-*d_6_*	[98]
*Silybum marianum* pericarp	silymarin	residual solvent signal (DMSO-*d_5_*)	DMSO-*d_6_*	[99]
*Stephania epigeae*	dicentrine, sinomenine	Dimethyl terephthalate	DMSO-*d_6_*	[100]
*Silybum marianum*	silymarin	residual solvent signal (DMSO-*d_5_*)	DMSO-*d_6_*	[101]
*Lepidium meyenii Walp.*	total macamides	1,3-dinitrobenzene	CDCl_3_	[102]
